# Supplementary material for: A multiscale approach for bridging the gap between potency, efficacy, and safety of small molecules directed at membrane proteins
Source: Sci Rep. 2021 Aug 16;11:16580. doi: 10.1038/s41598-021-96217-7 (PMC8368179; doi:10.1038/s41598-021-96217-7)
Supplement: Supplementary file 1 — Supplementary Information. [file 41598_2021_96217_MOESM1_ESM.pdf]

# Supplementary Information for

## A multiscale approach for bridging the gap between potency, efficacy, and safety of small molecules directed at membrane proteins

Rodrigo Aguayo-Ortiz,[a,d,1] Jeffery Creech,[a,b],[1] Eric N. Jiménez-Vázquez,[a] Guadalupe Guerrero-Serna,[a] Nulang Wang,[a] Andre Monteiro da Rocha,[a,b] Todd J. Herron,[a,b,c] and L. Michel Espinoza-Fonseca[a,*]

[a]Center for Arrhythmia Research, Department of Internal Medicine, Division of Cardiovascular Medicine, University of Michigan, Ann Arbor, MI 48109, USA.

[b]Frankel Cardiovascular Regeneration Core Laboratory, University of Michigan, Ann Arbor, MI 48109, USA.

[c]CARTOX, Inc., 56655 Grand River Ave., PO Box 304, New Hudson, MI 48165, USA.

[d]Departamento de Farmacia, Facultad de Química, Universidad Nacional Autónoma de México, Mexico City 04510, Mexico

*To whom correspondence should be addressed; Email: lmef@umich.edu

[1]R. A.-O. and J.C. contributed equally to this work.

**This PDF file includes:**

Supplementary Methods
Figures S1 and S2
Table S1

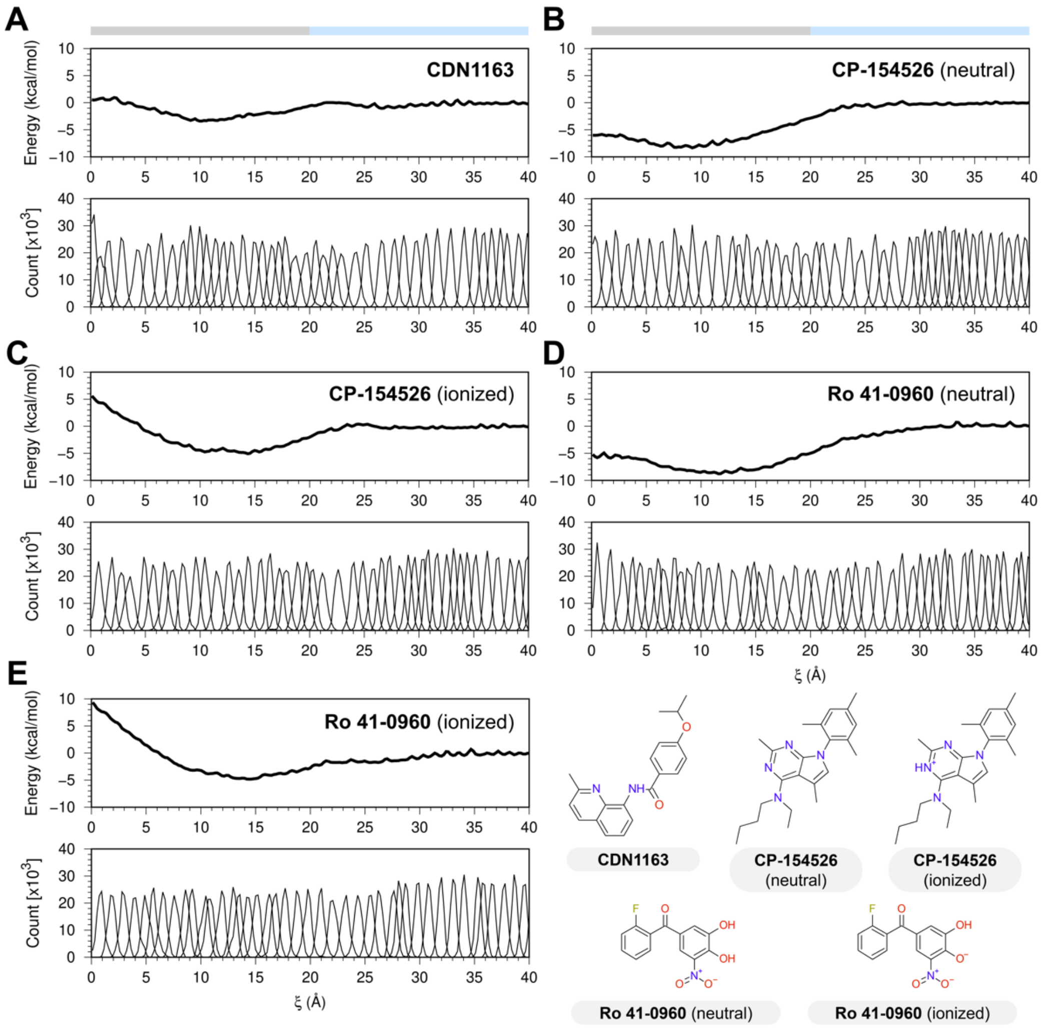


**Fig. S1. Umbrella sampling analysis.** Potential of mean force (PMF) profile (top) and histograms (bottom) of (A) CDN1163, (B, C) CP-154526, and (D, E) Ro 41-0960 through the POPC lipid bilayer along the reaction coordinate (ξ). The lower right section shows the chemical structures of the compounds and the protonation states used during the study.

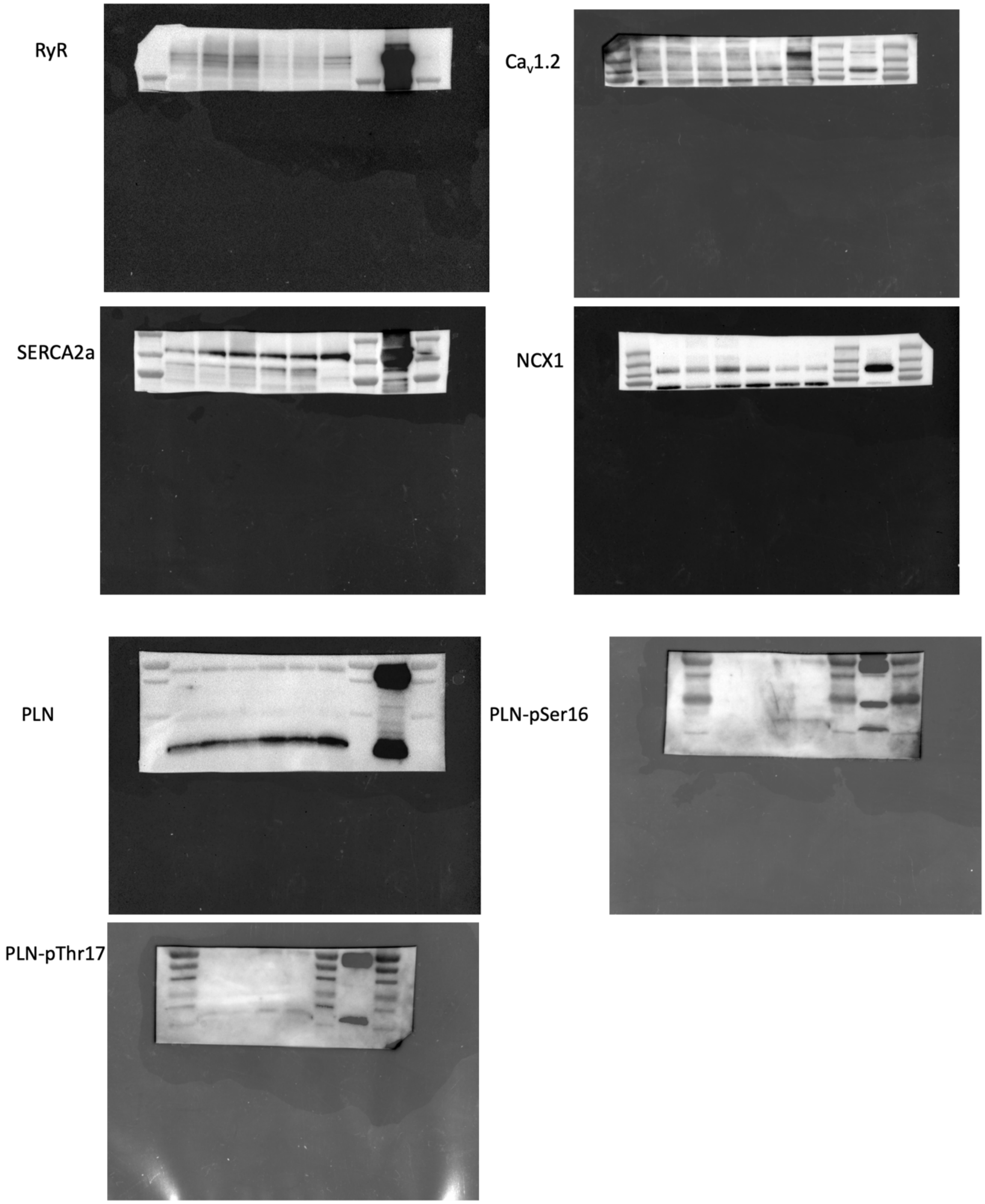


**Fig. S2.** Original images (tiff format) exported from the Image Lab software (Bio-Rad) obtained by the scanning of blots. For the final Figure 5 in the main text, we crop the black/grey area of each image and adjust the size in a way that all blots have the same length. In some cases, the levels were adjusted so that the bands become more apparent; the whole horizontal lines of bands were adjusted at the same time.

Table S1. Antibodies used to detect and quantify expression of $Ca^{2+}$-handling proteins in iPSC-derived cardiac cells

| Primary antibodies | Vendor/Supplier | Catalog # | Dilution |
|---|---|---|---|
| GAPDH | Sigma | G-8785 | 1:2000 |
| Serca2a | ThermoFisher | MA3-919 | 1:500 |
| Phospholamban | Badrilla | A010-14 | 1:5000 |
| Phospho-phospholamban (PLN-Ser16) | Badrilla | A010-12AP | 1:2500 |
| Phospho-phospholamban (PLN-Thr17) | Badrilla | A010-13AP | 1:1000 |
| Ryanodine Receptor (all RYR) | ThermoFisher | MA3-925 | 1:500 |
| Cav1.2 | Alomone | ACC-003 | 1:250 |
| NCX1 | Swant | R3F1 | 1:500 |
| **Secondary antibodies** | | | |
| Goat anti-mouse-HRP | Jackson ImmunoResearch | 115-035-166 | 1:1000<br>1:5000 for GAPDH |
| Goat anti-rabbit-HRP | Jackson ImmunoResearch | 111-035-144 | 1:1000 |
